# Effect of Low or High Pressure Alveolar Recruitment Maneuver on Postoperative Pain and Quality of Recovery in Patients with Obesity Undergoing Laparoscopic Sleeve Gastrectomy

**DOI:** 10.3390/jpm12101550

**Published:** 2022-09-21

**Authors:** Youn-Yi Jo, Seong-Min Kim, Dongchul Lee, Yong-Beom Kim, Jeongho Cha, Hyun-Jeong Kwak

**Affiliations:** 1 Department of Anesthesiology and Pain Medicine, College of Medicine, Gachon University, Gil Hospital, Incheon 21565, Korea; 2 Department of Surgery, College of Medicine, Gachon University, Gil Hospital, Incheon 21565, Korea

**Keywords:** laparoscopic bariatric surgery, alveolar recruitment maneuver, postoperative pain

## Abstract

Background: This study aimed to evaluate whether a low- or high-pressure alveolar recruitment maneuver (ARM) might reduce postoperative pain and improve the quality of recovery after laparoscopic bariatric surgery. Methods: 90 patients with a body mass index > 30 kg/m^2^ scheduled for laparoscopic sleeve gastrectomy were randomly assigned to control (*n* = 30), low ARM (*n* = 30), or high ARM groups (*n* = 30). For the low and high ARM groups, ARM was repeated five times to hold the peak airway pressure at 30 cmH_2_O and 60 cmH_2_O for 5 s, respectively, before removal of the trocar. Conventional methods to reduce post-laparoscopic pain, such as intraperitoneal saline irrigation, hemovac drainage, and gentle abdominal compression were performed in all patients, regardless of the assigned group. Results: Shoulder and surgical site pain scores 24 h postoperatively and rescue meperidine requirement were similar between the groups (*p* = 0.141, 0.101, and 0.82, respectively). The quality of recovery 40 (QoR40) score 24 h postoperatively was similar between the groups (*p* = 0.755). Postoperative pulmonary complications were similar between the groups (*p* = 0.124). Conclusion: Application of a low- or high-pressure ARM in addition to conventional methods to remove remnant peritoneal CO_2_ gas did not reduce postoperative shoulder or surgical site pain or improve the quality of recovery after laparoscopic sleeve gastrectomy.

## 1. Introduction

Laparoscopic surgery has become the mainstream abdominal surgery owing to its advantages of being less invasive and offering faster recovery for patients. However, although the pain at the surgical site is less than that of laparotomy, a large number of patients suffer from shoulder pain after surgery. The incidence of shoulder pain after laparoscopic surgery is reported to be high, at 60–80%, and it is known that the intensity is most severe on the first day after surgery [1,2,3]. In a previous study on gynecological laparoscopy, 80% of patients complained of shoulder pain after surgery, and the use of analgesics did not relieve the shoulder pain as effectively as pain at the surgical site [2]. Shoulder pain after laparoscopy is thought to be referred pain caused by the connection of the phrenic nerve (C3-5) that innervates the pleural surface of the diaphragm and the supraclavicular nerve (C3-4), which is responsible for the sensory input of the shoulder’s acromion process [4,5]. Shoulder pain may occur due to diaphragmatic irritation by carbon dioxide (CO_2_) insufflation or blood collection, extensive diaphragmatic stretching, or surgical manipulation to touch the diaphragm during laparoscopic surgery [5].

The alveolar recruitment maneuver (ARM) activates alveolar mobilization by temporarily applying high airway pressure as part of a lung-protection strategy. Studies have shown that the application of ARMs at the end of surgery can promote the emission of remnant CO_2_ in the peritoneal cavity, thus, reducing shoulder pain after laparoscopy [6,7]. A randomized clinical trial of laparoscopic surgery reported that applying ARM at a maximal airway pressure of 40 cmH_2_O could significantly reduce postoperative shoulder and upper abdominal pain at 12 and 24 h after surgery compared with the control group [6]. In another study comparing the ARM effect in patients undergoing laparoscopic gynecologic surgery using maximal airway pressure of 40 cmH_2_O and 60 cmH_2_O, both ARM methods significantly reduced shoulder pain compared with the control group [7]. In addition, a meta-analysis involving 571 patients reported that ARMs were effective in both reducing shoulder and upper abdominal pain after laparoscopic procedures [5].

In laparoscopic bariatric surgery, shoulder pain after surgery is reported to be close to 70% [8]. However, few studies have been conducted to determine whether ARMs relieve postoperative pain in laparoscopic bariatric surgery. Performing ARM at the end of laparoscopy could reduce postoperative pain and improve the quality of recovery after anesthesia in patients with obesity. This study aimed to evaluate the effect of ARM using 30 cmH_2_O and 60 cmH_2_O of peak airway pressure on postoperative shoulder and abdominal pain and the quality of recovery score after anesthesia in patients undergoing laparoscopic bariatric surgery.

## 2. Materials and Methods

Permission from the Institutional Review Board of Gachon University Gil Hospital was obtained prior to proceeding with this study (GDIRB2019-360) which was registered at clinicaltrial.gov (NCT04259918) prior to patient recruitment. Written informed consent was obtained from all participants. This study included 90 patients scheduled for elective laparoscopic sleeve gastrectomy and aged 20–65 years, with an American Society of Anesthesiologists physical status of 2 and a body mass index (BMI) over 30 kg/m^2^. Patients with a history of uncontrolled respiratory and/or cardiovascular morbidity, restrictive pulmonary disease, uncontrolled metabolic disorders, cerebrovascular disease, cognitive impairment, chronic pain, or those taking painkillers and/or steroids were excluded. Patients undergoing combined laparoscopic sleeve gastrectomy and hiatal hernia repairs were excluded. Patients were randomly assigned to a control group (*n* = 30), a low ARM group (*n* = 30), or a high ARM group (*n* = 30) using a randomized list generated by Excel 2017 (Microsoft Office, Redmond, WA, USA) without stratification. Patients, care providers, and the postoperative outcome assessor were unaware of the group assignment except anesthesiologists conducting the study.

No sedatives or analgesics were administered as premedication. Standard anesthetic monitoring, including a non-invasive blood pressure monitor, electrocardiogram (ECG), pulse oximeter, and bispectral index (BIS) were employed in the operating room. For anesthetic induction, lidocaine, propofol, remifentanil, and rocuronium were administered, and for maintenance of anesthesia, sevoflurane and remifentanil were used while targeting a BIS of 40–60. The mechanical ventilator setting was s volume-controlled mode with an inspired oxygen fraction (FiO_2_) of 0.6, a tidal volume of 8 mL/kg of ideal body weight [0.919 × (height in cm − 152.4) + 50 for men, or 45.5 for women], an inspiratory to expiratory (I/E) ratio of 1:2, a positive end-expiratory pressure (PEEP) of 5 cmH_2_O, and a respiratory rate adjusted to a target end-tidal carbon dioxide tension (ETCO_2_) of 40 ± 5 mmHg. Carbon dioxide insufflation for pneumoperitoneum at 16–18 mmHg and a 60° reverse-Trendelenburg position was adopted.

All patients underwent an intraperitoneal saline irrigation before trocar removal, and a hemovac drainage tube was placed through the trocar site. At the end of the surgery, the abdomen was gently compressed to remove CO_2_ gas. In the control group, initial ventilator settings were maintained. With the trocar open before removal of the trocar, ARM was repeated five times for the low and high ARM groups, maintaining the peak airway pressure at 30 cmH_2_O and 60 cmH_2_O for 5 s, respectively. When the systolic blood pressure dropped below 90 mm Hg or mean arterial pressure below 80% of the baseline value, phenylephrine 100 µg i.v. or ephedrine 5 mg i.v. was administered as appropriate. Patient-controlled analgesia (PCA) was provided for 48 h with fentanyl 0.15 µg × [ideal body weight + (0.4 × excess weight)]/cc in normal saline 100 mL, (basal infusion rate, 2 mL/h, 0.5 mL intermittent bolus with a 15 min lock-out interval). Ramosetron 0.3 mg was administered intravenously before the end of surgery to prevent postoperative nausea and vomiting.

Mean arterial pressure, heart rate, oxygen saturation, and vasopressor use were recorded pre-induction immediately before ARM, and 1, 3, and 5 min after ARM during laparoscopy. In the postanesthetic care unit (PACU), overall postoperative pain was assessed using an 11-point numerical rating scale (NRS) (0 = no pain, 10 = the worst imaginable pain) at 30 min after arrival. Because it was difficult for patients to express the pain area in detail immediately after anesthesia emergence, the severity of overall pain was investigated without dividing it into shoulder and abdominal pain (surgical site pain). The severity of nausea, frequency of vomiting, rescue antiemetics, and requirement for rescue fentanyl were assessed. A rescue fentanyl 50 µg intravenous bolus was administered when the NRS pain score was greater than five points or as needed by the patient.

At 24 h after surgery, the pain score (NRS) of postoperative shoulder pain and abdominal pain, total infused PCA volume for 24 h, and rescue analgesic requirement were evaluated. The quality of recovery (QoR) score was assessed using the 40-item multidimensional questionnaire [9] by one of the investigators. This questionnaire pertains to five dimensions of the recovery profile, as follows: physical comfort (12 questions), emotional state (9 questions), psychological support (7 questions), physical independence (5 questions), and pain (7 questions) [9]. Each question is equivalent to five points, and the global score ranges from 40 to 200 [9].

The low and high ARM groups might have similar effectiveness in reducing post-laparoscopic shoulder pain compared to the control group, based on a previous study [7]. The sample size was calculated based on earlier data, which reported that the standard deviation of the post-laparoscopic shoulder pain score was 1.3 at 24 h after the surgery [6,7]. To detect a one-point difference between the groups, 30 patients per group would be required when an alpha error of 0.05 and a 1-beta of 80% were set with 10% of possible drop-out rate.

In this study, SPSS software ver. 22.0 (SPSS Inc., Chicago, IL, USA) was used for the statistical analysis. The results are presented as mean [95% confidence interval], median [interquartile range], or number of patients. The Kolmogorov–Smirnov test was used to assess the normality of the continuous variable distribution. One-way ANOVA or a Kruskal–Wallis test with Bonferroni correction was used according to the normality test. Categorical variables were analyzed using the chi-squared test. A *p*-value < 0.05 was considered statistically significant except for in pairwise comparisons between the two groups. For Bonferroni-corrected significance level for multiple pairwise comparisons, a *p*-value < 0.05/3, was considered statistically significant.

## 3. Results

### 3.1. Participants

**Figure 1:**
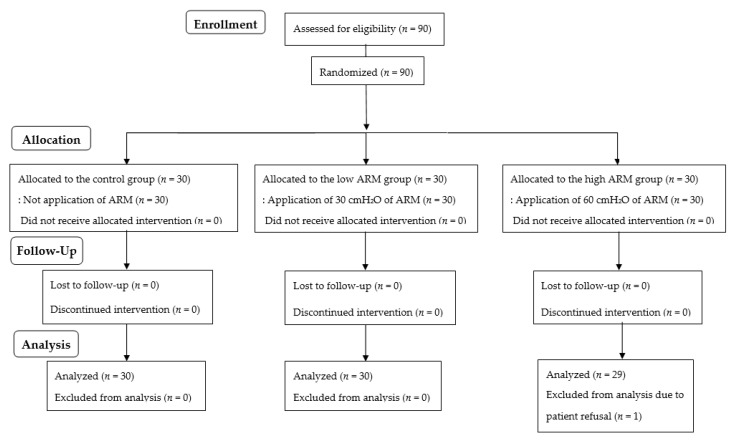
Flow diagram of patient allocation.

Among the 90 enrolled patients, one in the high ARM group was excluded from the analysis because of patient refusal (Figure 1). The patient characteristics and perioperative data are presented in Table 1. Perioperative data, including pneumoperitoneum time, were similar between groups.

### 3.2. Intraoperative Hemodynamic Changes

The intraoperative hemodynamic changes and vasopressor requirements are shown in Table 2. There were no intergroup differences in mean arterial pressure, heart rate, SpO_2_, or the frequency of vasopressor use during surgery.

### 3.3. PACU Data

The data in the PACU are presented in Table 3. The overall pain score and rescue fentanyl requirement were similar between the groups (*p* = 318 and 0.993, respectively). The severity of postoperative nausea and the frequency of vomiting and rescue antiemetic use were similar between the groups (*p* = 0.245, 0.835, and 0.469, respectively).

### 3.4. Postoperative Pain and QoR40 Scores

Postoperative pain and QoR scores during the postoperative 24 h are demonstrated in Table 4. There were no intergroup differences in post-laparoscopic shoulder and abdominal pain (*p* = 0.141 and 0.101, respectively). The number of patients with shoulder pain that was more severe than abdominal pain was not statistically significant (*p* = 0.196). The rescue meperidine requirement and total infused PCA volume for 24 h were also similar between the groups (*p* = 0.820 and 0.591, respectively). The total score and each dimension score of QoR at 24 h postoperatively were similar between the groups. Postoperative pulmonary complications, including atelectasis and pulmonary edema, were observed in 4 patients in the control group, 2 patients in the low ARM group, and 0 in the high ARM group, without statistically significant difference (*p* = 0.124). There were no patients whose symptoms were severe enough to delay discharge or require additional treatment, and the length of hospital stay did not differ between the groups (*p* = 0.339).

## 4. Discussion

In this prospective study, additional application of ARM with a peak airway pressure of 30 cmH_2_O or 60 cmH_2_O at the end of laparoscopy was found to neither reduce postoperative shoulder nor abdominal pain, and did not improve QoR after anesthesia, in patients undergoing laparoscopic bariatric surgery. 

A previous study comparing ARM with multiple levels of peak inspiratory pressure with no ARM demonstrated that all levels of ARM had a beneficial effect in improving post-laparoscopic shoulder pain [7]. In addition, in a recent meta-analysis involving 571 patients, wherein ARM was applied in 291 (51%) patients and conventional treatments, such as passive evacuation of CO_2_ gas were employed in 280 (49%) patients, ARM application significantly decreased shoulder pain for postoperative patients at 48 h [10]. Another meta-analysis reported that ARM could reduce the severity of shoulder pain and decrease the requirement of analgesics after laparoscopy for 24 h postoperatively [11]. 

Contrary to the aforementioned studies in patients without obesity, the current study on patients with obesity did not demonstrate any beneficial effects of ARM in decreasing postoperative shoulder or abdominal pain or improving QoR, even when low or high peak airway pressure was applied. In a risk analysis of shoulder pain after laparoscopic infertility surgery, postoperative shoulder pain showed a negative correlation with BMI (odds ratio = 0.815; 95% confidence interval 0.767–0.866), and the pain score was significantly higher in BMI of 30 kg/m^2^ or less than in BMI > 30 kg/m^2^ [4]. Patients without obesity were observed to have enough space in the upper abdomen to store CO_2_ gas, so that the anatomy of the liver and diaphragm could be clearly seen, whereas patients with obesity had less space for storing gas because the anatomy in the same location was covered by the omentum [4]. As such, since postoperative pain related to CO_2_ pneumoperitoneum occurs less in patients with obesity, it can be assumed that the pain-reducing effect of ARM was also attenuated in this study. 

Several strategies have been suggested to reduce the need for post-laparoscopic surgery. Intraperitoneal saline irrigation during laparoscopic cholecystectomy reduced the concentration of CO_2_ remaining in the abdominal cavity, which may decrease postoperative pain on the day of surgery; however, the use of rescue analgesics did not decrease [12]. Acetazolamide, a carbonic anhydrase inhibitor, can also effectively reduce post-laparoscopic referred pain by promoting the diffusion of intraperitoneal CO_2_ into the blood stream [13]. From these results, it can be concluded that CO_2_ remaining in the abdominal cavity causes pain after laparoscopic surgery. Another study showed that intraperitoneal saline irrigation was superior to preoperative oral acetazolamide administration in reducing post-laparoscopic pain and rescue fentanyl use [14]. Hemovac drainage through the trocar site may improve shoulder pain after laparoscopic gynecologic surgery when compared with postoperative deep breathing or controls [15], and intraperitoneal drainage has been shown to reduce the incidence and severity of shoulder pain during the early postoperative period [11]. Gentle abdominal compression during trocar opening is an easy and safe procedure for expelling residual intraperitoneal CO_2_ [16]. Rettenmaier et al. reported that the postoperative pain score declined steadily at 12, 24, and 48 h postoperatively in patients who underwent abdominal compression at the end of the laparoscopic procedure [16]. 

In this study, conventional methods to reduce post-laparoscopic pain, such as intraperitoneal saline irrigation, hemovac drainage through the trocar site, and gentle abdominal compression during trocar opening, were performed in all patients, regardless of the assigned group. It can be considered that these procedures not only reduced the postoperative pain score of the control group, but also reduced the pain score differences between the control and the ARM groups. The reason for reducing postoperative pain with ARM may be to evacuate as much CO_2_ gas as possible, and how this is achieved does not seem to matter. There are studies showing that ARM reduces postoperative pain in laparoscopic surgery for non-obese patients [3,6,7]. However, because it is not known whether ARM alone without other conventional methods would be effective in obese patients, additional research may be needed. 

The QoR questionnaire, a widely used self-report questionnaire, was used to evaluate the quality of postoperative recovery, and includes emotional and psychological status as well as the most uncomfortable postoperative situations, such as pain, nausea, vomiting, and possible breathing problems [9]. In this study, differences between the groups could not be determined using this questionnaire. In addition, there were no clinically or statistically significant differences in postoperative pulmonary complications. This result is consistent with that of a previous study that reported that the application of ARM did not improve functional residual capacity and arterial oxygenation after laparoscopic gastric bypass in patients with morbid obesity [17]. Meanwhile, the median NRS of nausea was three times higher in the control group than in the high ARM group, although the difference was not statistically significant. The lack of statistical significance may be due to the insufficient number of patients, so a large-scale studies of the relationship between nausea and ARM might be needed.

## 5. Conclusions

In conclusion, the application of a low- or high-pressure ARM in addition to conventional methods to remove remnant peritoneal CO_2_ gas did not reduce postoperative shoulder or surgical site pain or improve the quality of recovery after laparoscopic sleeve gastrectomy in patients with obesity. Thus, ARM application may have no additional benefit after laparoscopic bariatric surgery if safe and effective methods to remove remnant CO_2_ from the abdominal cavity are included. 

## Figures and Tables

**Table 1:** Patient characteristics and perioperative data.

	Control (*n* = 30)	Low ARM (*n* = 30)	High ARM (*n* = 29)	*p* Value
Age (years)	36 [33–39]	35 [32–39]	33 [30–36]	0.530
Gender (M/F)	3/27	5/25	4/25	0.750
Body mass index (kg/m^2^)	38 [36–41]	38 [36–40]	37 [36–39]	0.826
Diabetes mellitus (*n*)	12	8	10	0.547
Hypertension (*n*)	16	8	13	0.101
Anesthesia time (min)	143 [125–161]	153 [128–178]	140 [124–156]	0.615
Operation time (min)	115 [97–132]	122 [99–146]	109 [94–125]	0.617
Pneumoperitoneum time (min)	89 [73–105]	97 [74–121]	89 [68–93]	0.432

Values are presented as mean [95% confidence interval] or the number of patients. Control, not applying alveolar recruitment maneuver (ARM); low and high ARM, applying peak airway pressure 30 cmH_2_O and 60 cmH_2_O of ARM, respectively.

**Table 2:** Intraoperative hemodynamic changes and vasopressor requirements.

Variables	Group	Baseline	Before ARM	ARM 1 min	ARM 3 min	ARM 5 min
Mean BP (mmHg)	Control	101 [94–107]	85 [81–89]	87 [84–91]	86 [83–90]	87 [82–91]
	Low ARM	97 [91–102]	91 [84–98]	90 [83–96]	88 [83–94]	89 [83–95]
	High ARM	100 [93–107]	88 [82–94]	87 [80–94]	87 [81–93]	87 [81–92]
Heart rate (beats/min)	Control	80 [76–85]	84 [79–89]	82 [77–87]	82 [77–87]	83 [78–88]
	Low ARM	77 [73–81]	83 [78–88]	81 [75–86]	79 [75–84]	80 [75–85]
	High ARM	82 [78–87]	78 [74–83]	81 [76–85]	80 [76–85]	81 [77–85]
SpO_2_ (%)	Control	99 [99–100]	99 [98–99]	99 [99–99]	99 [98–99]	99 [98–99]
	Low ARM	99 [99–100]	99 [99–100]	100 [99–100]	100 [99–100]	100 [99–100]
	High ARM	99 [98–99]	99 [98–99]	99 [99–100]	99 [99–100]	99 [99–100]
Vasopressor use (*n*)	Control	1	1	0	0	0
	Low ARM	1	1	0	0	0
	High ARM	1	0	0	0	0

Values are presented as mean [95% confidence interval]. Control, not applying alveolar recruitment maneuver (ARM); low and high ARM, applying peak airway pressure 30 cmH_2_O and 60 cmH_2_O of ARM, respectively; Baseline, before anesthetic induction; Before ARM, ARM 1 min, ARM 3 min, ARM 5 min, immediately before alveolar recruitment maneuver (ARM), 1, 3, and 5 min after ARM at the end of the pneumoperitoneum; mean BP, mean blood pressure.

**Table 3:** Data in the postanesthetic care unit.

	Control (*n* = 30)	Low ARM (*n* = 30)	High ARM (*n* = 29)	*p* Value
Pain score (NRS)	5 [4–6]	5 [4–7]	6 [3–7]	0.318
Rescue fentanyl (µg)	50 [0–100]	50 [0–100]	50 [0–88]	0.993
Nausea (*n*)	16	11	9	0.191
Nausea (NRS)	3 [0–5]	1 [0–5]	1 [0–4]	0.245
Vomiting (*n*)	2	2	3	0.833
Rescue antiemetics use (*n*)	8	6	4	0.469

Values are presented as median [interquartile range], or number of patients. Control, not applying alveolar recruitment maneuver (ARM); low and high ARM, applying peak airway pressure 30 cmH_2_O and 60 cmH_2_O of ARM, respectively; NRS, 11-points numerical rating scale (0 = no pain, 10 = the worst imaginable pain).

**Table 4:** Postoperative data and the quality of recovery (QoR) score at postoperative 24 h.

	Control (*n* = 30)	Low ARM (*n* = 30)	High ARM (*n* = 29)	*p* Value
Shoulder pain (NRS)	3 [1–3]	2 [0–3]	2 [0–2]	0.141
Abdominal pain (NRS)	3 [2–5]	3 [3–5]	3 [2–3]	0.101
Shoulder pain > abdominal pain (*n*)	8	3	4	0.196
Rescue meperidine (mg)	50 [25–50]	50 [25–50]	50 [25–50]	0.820
Infused PCA (mL)	55 [40–75]	64 [51–76]	60 [42–80]	0.591
Total QoR score	149 [138–160]	152 [143–160]	155 [142–167]	0.755
Physical comfort	46 [43–49]	46 [43–48]	46 [43–50]	0.935
Emotional state	36 [34–39]	36 [34–38]	37 [34–39]	0.904
Psychological support	27 [25–30]	28 [26–30]	28 [26–31]	0.818
Physical independence	14 [12–16]	14 [12–16]	16 [14–18]	0.374
Pain	27 [25–29]	28 [27–30]	28 [25–30]	0.589
Postoperative complications (*n*)	4	1	0	0.124
Hospital stay (day)	5 [5–6]	5 [5–7]	5 [5–7]	0.339

Values are presented as mean [95% confidence interval], median [interquartile range (IQR)], or number of patients. Control, not applying alveolar recruitment maneuver (ARM); low and high ARM, applying peak airway pressure 30 cmH_2_O and 60 cmH_2_O of ARM, respectively; NRS, 11-points numerical rating scale (0 = no pain, 10 = the worst imaginable pain); PCA, patient-controlled analgesia; QoR, quality of recovery questionnaire.

## Data Availability

Data is contained within the article.
